# Effects of Long-Term Pioglitazone Treatment on Peripheral and Central Markers of Aging

**DOI:** 10.1371/journal.pone.0010405

**Published:** 2010-04-29

**Authors:** Eric M. Blalock, Jeremiah T. Phelps, Tristano Pancani, James L. Searcy, Katie L. Anderson, John C. Gant, Jelena Popovic, Margarita G. Avdiushko, Don A. Cohen, Kuey-Chu Chen, Nada M. Porter, Olivier Thibault

**Affiliations:** 1 Department of Molecular and Biomedical Pharmacology, University of Kentucky Medical Center, Lexington, Kentucky, United States of America; 2 Department of Microbiology and Immunology, University of Kentucky Medical Center, Lexington, Kentucky, United States of America; Sapienza University of Rome, Italy

## Abstract

**Background:**

Thiazolidinediones (TZDs) activate peroxisome proliferator-activated receptor gamma (PPARγ) and are used clinically to help restore peripheral insulin sensitivity in Type 2 diabetes (T2DM). Interestingly, long-term treatment of mouse models of Alzheimer's disease (AD) with TZDs also has been shown to reduce several well-established brain biomarkers of AD including inflammation, oxidative stress and Aβ accumulation. While TZD's actions in AD models help to elucidate the mechanisms underlying their potentially beneficial effects in AD patients, little is known about the functional consequences of TZDs in animal models of normal aging. Because aging is a common risk factor for both AD and T2DM, we investigated whether the TZD, pioglitazone could alter brain aging under non-pathological conditions.

**Methods and Findings:**

We used the F344 rat model of aging, and monitored behavioral, electrophysiological, and molecular variables to assess the effects of pioglitazone (PIO-Actos® a TZD) on several peripheral (blood and liver) and central (hippocampal) biomarkers of aging. Starting at 3 months or 17 months of age, male rats were treated for 4–5 months with either a control or a PIO-containing diet (final dose approximately 2.3 mg/kg body weight/day). A significant reduction in the Ca^2+^-dependent afterhyperpolarization was seen in the aged animals, with no significant change in long-term potentiation maintenance or learning and memory performance. Blood insulin levels were unchanged with age, but significantly reduced by PIO. Finally, a combination of microarray analyses on hippocampal tissue and serum-based multiplex cytokine assays revealed that age-dependent inflammatory increases were not reversed by PIO.

**Conclusions:**

While current research efforts continue to identify the underlying processes responsible for the progressive decline in cognitive function seen during normal aging, available medical treatments are still very limited. Because TZDs have been shown to have benefits in age-related conditions such as T2DM and AD, our study was aimed at elucidating PIO's potentially beneficial actions in normal aging. Using a clinically-relevant dose and delivery method, long-term PIO treatment was able to blunt several indices of aging but apparently affected neither age-related cognitive decline nor peripheral/central age-related increases in inflammatory signaling.

## Introduction

Adjunct therapy against type 2 diabetes mellitus (T2DM) with thiazolidinediones (TZDs) is on the rise, with increasing numbers of patients prescribed the TZDs rosiglitazone (ROSI, Avandia®) or pioglitazone (PIO, Actos®). These agents are in the top 50 prescribed drugs in North America, and together account for approximately 20 million prescriptions (2008 data, rxlist.com). Results from over two decades of studies have shown that untreated T2DM can negatively impact brain function. Depending on the severity and the duration of the disease, as well as on the age of the individual, the condition is associated with varying degrees of cognitive deficits, motor dysfunction, and depression [1], [2], [3], [4], [5], [6], [7]. While aging worsens the impact of diabetes on cognitive function, it is not clear how diabetes and accompanying peripheral metabolic dysregulation exacerbate this process. Proposed mechanisms underlying cognitive decline when aging and diabetes coexist include insulin resistance, vascular disease, and inflammation resulting from the release of adipose tissue-derived cytokines. In clinical and animal studies, the brain, and the hippocampus in particular, appear sensitive to peripheral cytokine levels or metabolic stressors [8], [9], [10], [11], with enhanced sensitivity seen in aged animals [8]. Given the role of the hippocampus in memory acquisition, processing and consolidation (reviewed in [12], [13]), inflammation within the structure likely contributes to memory and cognitive deficits with age and/or AD. Nevertheless, almost nothing is known about the mechanisms through which peripheral metabolic dysregulation as those seen in T2DM impact hippocampal function and cognition.

TZDs are best known for their peripheral actions, where these synthetic PPARγ agonists selectively bind nuclear receptors and enhance lipid accumulation in adipocytes, thereby helping to decrease free fatty acid and lipid levels in plasma [14], [15], [16], [17], [18]. This mechanism helps to reestablish insulin sensitivity in T2DM by working on fat, liver, and muscle tissues. Additionally, TZDs are compounds with significant anti-inflammatory actions [19], [20]. Recent evidence indicates that TZDs can have beneficial central effects. In particular, ROSI was shown to improve cognition and verbal memory in patients with mild cognitive impairment (MCI) [21]. Further, a recent preliminary study on patients diagnosed with MCI and diabetes also reported improved cognition following 6-months of PIO [22]. Finally, AD patients lacking the ApoEε4 allele appear to be selectively sensitive to the beneficial effects of chronic treatment with ROSI [23]. The mechanisms underlying these effects in humans are not clear, but are likely to reflect changes in inflammation, vascular function, insulin and/or glucose levels, energy metabolism or beta amyloid clearance. Whether these improvements are due to changes in the periphery, direct effects in the brain, or some combination, is still unknown. In AD animal models (APPV717I, Tg2576, 3×TG), TZDs have been shown to decrease Aβ deposition [24], [25], [26], [27] (but see [28]), inflammation [24], [29], [30], and oxidative stress [28].

The predominant information we have regarding the beneficial effects of TZDs in the brain comes from clinical studies in AD patients [21], [23]. However, given that most patients with diabetes do not have AD, at least in the earlier stages of the disease, it seems important to determine what the potential effects of these drugs are in the context of normal aging, or in the absence of clinically defined cognitive deficits. Compared to research conducted in AD models, however, little is known about the functional consequences of TZDs on cognition in animal models of normal aging. Therefore, the present studies were undertaken to determine whether PIO, the more brain permeant TZD [26], confers significant benefits within the context of normal brain aging. Further, because of our prior work identifying new roles of ROSI and PIO in cultured neurons [31], and the work of others suggesting that targets of TZD actions may include Ca^2+^-mediated pathways in the brain [32], [33], [34], we investigated select biomarkers of aging including the Ca^2+^-dependent afterhyperpolarization (AHP), long-term potentiation (LTP), and hippocampal-dependent spatial memory. Other examined variables in the brain and in the periphery included inflammatory cytokine levels, hippocampal gene signatures, and insulin signaling. Our results suggest that at the dose and duration tested, PIO caused expected beneficial effects including reduced peripheral insulin and lipid marker levels, and reduced a central biomarker of aging, namely the AHP. However, other major markers of aging, including increased inflammatory signaling (based on cytokine array measures in the periphery and microarray measures in the hippocampus), impaired cognition, and altered synaptic plasticity, were not altered with PIO treatment.

## Methods

### Ethics Statement

All procedures were carried out under a University of Kentucky IACUC approved protocol and are in accordance with NIH guidelines for the care and use of laboratory animals.

### Animals

Thirty-six male F344/NIA rats were purchased (Harlan, Indianapolis, IN) in 2 groups of 18 with a 6 week stagger between groups. All animals were fed TD94045 diet (Harlan Teklad, Madison, WI) for one week prior to initiation of PIO or control diets. To limit potential cohort effects across two separate animal purchases, treatment groups were balanced across both cohorts. Each group consisted of 8 young (3 months old), and 10 aged (17 months old) animals. Twelve animals from each group were assigned to either the young control (YC, n = 4), young PIO (YP, n = 4), or aged control (AC, n = 4) treatment groups, and the remaining six animals were placed in the aged PIO (AP) group. In all, the study was comprised of 8 YC, 8 YP, 8 AC, and 12 AP animals. Animals were maintained on the diets for 15–20 weeks, and were 7–8 months old, and 21–22 months old at the time of study completion. Five aged animals died in the course of the study. One AP animal had to be euthanized because of an unresolved mandibular/eye infection, and two AP animals stopped eating, lost considerable weight and were euthanized. Based on two gross necropsy reports, another AP animal died of chronic renal failure, and 1 AC animals died of granular lymphocytic leukemia, both major causes of mortality in the aging F344 [35]. The thirty one remaining animals were active, well-groomed and appeared healthy, and were used for behavioral and electrophysiology studies.

### Blood collections and analysis

Over the course of the study, three *in vivo* glucose measures were taken. Animals were placed in a decapicone® restraint (Braintree Scientific, Braintree, MD) while their tails were washed with warm soapy water and dried under a heat lamp. The lateral tail vein was pricked with a 22 gauge needle and a FreeStyle Lite glucometer (Abott Diabetes Care Inc., Alameda, CA) was used to measure blood glucose levels (mg/dL). Trunk blood from twenty nine animals was collected at the time of hippocampal slice preparation (two samples were lost). Briefly, 2–3 mL was collected in a BD Vacutainer SSD centrifuge tube and allowed to clot at room temperature for one hour. To collect serum, samples were centrifuged at 4000 rpm for 10 min. Half of the serum was sent on dry ice for standard chemistry panel analysis (Comparative Pathology Laboratory, University of California Davis, CA). The remaining serum was frozen (−80°C) and later used to monitor insulin concentrations using the manufacturer's protocol for an ELISA-based assay (Millipore, Billerica, MA) and our bioassay reader (HTS plus 7000, Perkin Elmer, Wellesley, MA), as well as to monitor for the presence of three proinflammatory cytokines using a Multiplex Bio Assay Analyzer (Millipore).

### Insulin Receptor signaling

Liver and brain cortices were used to quantify total and phosphorylated insulin receptor levels according to the manufacturer's protocol (Calbiochem, San Diego, CA), and using duplicates for each sample. Frozen samples were removed from the −80°C freezer and thawed on ice. After a 2 min homogenization period in PBS followed by centrifugation at 300 rpm for 5 min, the pellet was resuspended in Cell Extraction Buffer (BioSource FNN0011) and left to lyse for 30 min with vortexing every 10 min. At the end of this process the suspension was centrifuged at 14K rpm for 10 min and the protein content in the supernatant was determined using a Bradford assay. Detection of the phosphorylated insulin receptor (IR) was accomplished following the Phosphodetect ELISA kit protocol (CBA038, Calbiochem). Total IR present in the samples was measured using IR β-subunit ELISA Kit (CBA039, Calbiochem). Briefly, samples containing the same concentration of total protein were incubated for 2 h in a 96 wells plate coated with IR β–subunit-specific monoclonal antibody. After washing, an antibody specific for IR phosphorylated at Tyr^1162/1163^ (CBA038) or specific for IR β-subunit (CBA039) was added (detection antibody). The excess detection antibody was removed after 1 hr and a horseradish peroxidase-conjugated antibody (anti-rabbit Ig-HRP) was added to the wells for 30 min. Following a final washing step to remove the excess anti-rabbit Ig-HRP, a substrate was added and absorbance was read at 450nm.

### Experimental diets

Pioglitazone (PIO-Actos®) was purchased through our DLAR facility, and was incorporated into standard, color-coded purified rodent diets (TD94045 Harlan Teklad). TD94045 was chosen to approximate the NIH31 diet fed to animals since adulthood (18.8% Kcal from protein, 63.9% Kcal from carbohydrates, and 17.2% Kcal from fat *vs.* 24% Kcal from protein, 62% Kcal from carbohydrates and 14% Kcal from fat in the NIH31). Because of differences in animal weights and food consumption, two PIO diets were used, one formulated at 84 ppm for older animals and another, formulated at 37 ppm for younger animals. Final PIO dosages based on animal food consumption and body weight (each measured 3 times a week throughout the course of the study) were ∼2.3 mg/Kg/day. Actos® is available in 15–45 mg tablets and in humans, serum concentrations following a single 30 mg oral dose reach approximately 1 µg/mL [36], and following a 10 day treatment with once a day 45 mg oral dosing regimen, peak plasma level was measured at 1.6 µg/mL, as reported in The pharmacological basis of therapeutics [37]. The dose used here (∼2.3 mg/Kg/day), is relatively low compared to other reports in animals, and we estimate steady state blood PIO levels at approximately 1.3 µg/mL. This is based on published human clearance values for PIO (1.2 mL/min/Kg) given that the pharmacokinetic properties of PIO in rodents are not available.

### Electrophysiology, AHP and LTP

Electrophysiological data were recorded between 1.5 and 5 weeks after the end of the Morris water maze training to limit the impact of learning and arousal on transient (about one week [38]) hippocampal excitability changes, and because only a single animal could be monitored daily on the electrophysiology setup. Hippocampal slices taken from the medial half of the hippocampus were obtained according to previously published protocols [39], briefly, animals were anesthetized in a CO_2_ chamber prior to decapitation, hippocampi were removed and transverse slices prepared (350 µm in ice cold low-calcium artificial cerebrospinal fluid (ACSF) composed of (in mM): 128 NaCl, 1.25 KH_2_PO_4_, 10 Glucose, 26 NaHCO_3_, 3 KCl, 0.1 CaCl_2_, 2 MgCl_2_.) using a Vibratome® (series 3000, TPI, Saint Louis, MO). For AHP experiments, slices were then transferred to a heated (32°C) interface-type chamber, maintained in oxygenated (95% O_2_, 5% CO_2_) normal-calcium ACSF containing 2mM CaCl_2_ and 2mM MgCl2 for least 2 h prior to recording. For LTP experiments, a modified ACSF containing 2.5 mM CaCl_2_ and 1.3 mM MgCl_2_ was used.

### AHP experiments

Each hippocampal slice was placed in a recording chamber (RC22C, Warner Instruments, Co., Hamden, CT) and maintained in a continuous flow of oxygenated ACSF pre-heated at 32°C using a TC2Bip/HPRE2 in line heating system (Cell Micro Controls, Northfolk, VA). This setup was mounted on the stage of a Nikon E600FN inverted microscope. As previously described [39], cells were impaled with sharp microelectrodes filled with 2M KMeSO_4_ and 10mM HEPES, pH 7.4 (tip resistance 108.2±4.7 MΩ), pulled from borosilicate glass capillaries (World Precision Instruments, Sarasota, FL) using a P80 pipette puller (Sutter Instruments, Novato, CA). All experiments were performed in current clamp mode with bridge balance compensation and capacitance neutralization. Signal was digitized at 2 kHz and low-pass filtered at 1 kHz. Recordings of membrane input resistance (IR) were obtained in response to 800 ms, 200 pA hyperpolarizing current injections using an Axoclamp 2B amplifier (Molecular Devices, MDS, Toronto, Canada) while holding the cell at −70 mV. To generate an afterhyperpolarization (AHP) cells were held at −65 mV (baseline) and depolarized with a 100 ms current injection in order to generate three Na^+^ action potentials. AHPs were elicited every 30 s and at least 6 AHPs were averaged for each cell. The medium AHP (mAHP) was measured as the peak hyperpolarization immediately after the offset of the depolarizing current injection, the slow AHP (sAHP) was measured 800 ms after the end of the current injection. The AHP duration was measured from the end of the depolarizing step until return to baseline. Neurons with input resistance <40 MΩ, holding current >500 pA and action potential height <0 mV, were excluded from in this study. Data were acquired using pClamp 8.0 (Molecular Devices) software through a Digidata 1320A A/D converter (Molecular Devices), and quantification of potentials (e.g., amplitude and duration of AHPs) was obtained with Clampfit software (Molecular Devices).

### LTP experiments

Slices were recorded from within a heated and oxygenated interface-type chamber (32°C) after at least 2 h of recovery. Recording electrodes were 5–10 MΩ (filled with ACSF), and the stimulating electrode was made from twisted insulated stainless steel wire (A-M Systems, Inc. Everett, WA). Stimulation (baseline and LTP) was delivered through a pair of SD9K stimulators (Astro Med Inc., Grass Instr., Warwick, RI). During baseline and after LTP induction, stimulation rate was set to 0.33 Hz. LTP was elicited using a 2 s theta-burst pattern such that eight pulses at 100 Hz (50 ms each) were delivered at 5 Hz [40] in stratum radiatum. Stimulation intensity was set at 33% of the maximum response (determined from an *I/O* curve prior to LTP induction). This LTP induction protocol was chosen to accentuate the age-dependent decrease in LTP induction and maintenance [13], [41], [42], [43], [44], [45], [46]. For each slice, baseline EPSP slopes averaged across the 20 min prior to LTP induction were used to normalize EPSP slopes after LTP induction. Post-tetanic potentiation (PTP) was derived from EPSP slope measures taken immediately after LTP induction (2 min average) and LTP was derived from EPSP slopes averaged 25–30 min after tetanization (5 min average). A slice was removed from the analysis if the percent change in EPSP slope during the baseline period fluctuated more than 25% (up or down), or if the EPSP was contaminated with a spike following LTP induction.

### Morris Water Maze (MWM)

The maze (black circular pool, 190 cm in diameter) was placed equidistant (∼60 cm) to a continuous wall of black curtains hanging from the ceiling, making the environment relatively neutral. Three high contrast black and white cues (90 cm×90 cm, representing a circle, triangle and vertical lines), were placed on the curtains. Each day, the animals were placed in one of the four quadrants; this allowed the animal to learn to map the position of the escape platform relative to the cues on the curtain. Pool temperature was maintained at 25–26°C. One quadrant contained a 15 cm diameter escape platform covered with black neoprene for improved traction. Illumination in the room was set such that the Videomex-V water maze monitoring system (Columbus Instrument, Columbus, OH) could reliably monitor animal movements with no artifacts.

For all training days (days 1–4), three trials were run with animals placed in the pool for 60 s. During the early training days (1–2), animals that did not find the platform within the allotted 60 s were gently guided to the platform. All animals were allowed to stay on the platform for 60 s. Following this 60 s rest period, animals were taken to a drying cage outside the MWM enclosure for 45 s, and then returned to the MWM for a second trial. The intertrial interval was approximately 165 s, with ∼60 s of swimming and 105 s of rest. On day 1, three cue trials were run with animals released in the same quadrant for each trial. In these first trials, a hanging white cup was positioned over the platform (∼30 cm above the water surface), and the platform was set right at, or slightly above the water level, providing the animals with salient clues for a mean of escape. On the next 3 days of training (days 2–4) animals were placed in a different starting location along the periphery of the maze for each trial (3 trials/day), and the platform was submerged (∼2.5 cm below the water surface). Animals were never placed in the pool within the quadrant containing the platform. On the last day (day 5), a single 60 s probe trial was run with the platform removed. Animals were considered visually impaired if they failed to find the platform within the allotted 60 s on 2 out of 3 trials on day 1 (cue learning) , and on 3 out of 3 trials for learning days 3 and 4. Using this criterion, 6 aged animals, 3 AC and 3 AP, were excluded from the behavioral analysis.

### Microarrays

#### Microarray analysis

During preparation of hippocampal tissue for electrophysiology, dorsal and ventral quarters from both hippocampi were placed in RNAse-free Eppendorf tubes on dry ice, and transferred to a −80°C freezer until further use. For each animal (n = 7–8/group), this tissue was treated as a single sample. Each sample underwent RNA extraction, purification, and cDNA labeling separately, as described previously [47], [48], [49], [50], according to standard Affymetrix procedures. Labeled cDNA for each region from each subject was individually hybridized to an Affymetrix rat microarray (RAE230 2.0, 31099 probe sets). All arrays passed standard Affymetrix quality control: GAPDH 3′–5′ ratio 1.07±0.005, RawQ 2.73±0.02, Background noise 79.3±0.6. Scaling factor, based on target intensity of 500, YC: 0.95±0.03, YP: 0.91±0.04, AC: 0.92±0.04, AC: 0.90±0.04; as well as % Present- YC: 69.7±0.44, YP: 70.0±0.33, AC: 69.8±0.51, AP: 70.1±0.35 were not significantly different across treatment groups (two-way ANOVA p>0.4 for main effects of age, drug, and interaction). Visual inspection of residual sign images (Affy PLM [51]) revealed no major image defects.

The MAS5 probe level algorithm (Gene Expression Console v 1.1, Affymetrix) calculated signal intensity and presence/absence calls. Only unique probe sets/genes with ‘A’ grade annotation and >2 presence calls were retained for further analysis. Values were transferred to Excel (2007, Microsoft), Bioconductor [52], MultiExperiment Viewer (MEV, [53]) and the DAVID suite of bioinformatic tools [54] for subsequent analysis. All data are MIAME compliant and the raw data has been deposited in a MIAME compliant database (Gene Expression Omnibus - GEO accession #GSE20219).

### Proinflammatory Cytokine Analysis

Serum samples were analyzed by multiplex bead array using Milliplex rat cytokine kits (RCYTO-80K) according to procedures recommended by the manufacturer (Millipore). Just prior to analysis, frozen sera were thawed and maintained on ice throughout the assay setup. Briefly, all serum samples were diluted 1∶5 in sample diluent and were then incubated in duplicate overnight with capture beads specific for IL-1β, IL-6 and TNFα. Beads were subsequently washed and incubated for 2h with biotin-conjugated detection antibody and then for 30 min with streptavidin-phycoerythrin. Bead fluorescence was then analyzed on a Luminex 100 IS Multiplex Bio-Assay Analyzer. Cytokine concentrations were determined from standard curves of recombinant rat cytokines in which 4-parameter logistic curve fitting analysis was used. All cytokines are reported as pg/ml ± S.D.

### Statistics

For all electrophysiological measures presented here, outliers were removed based on the 2 SD rule. For main effects of age or treatment on these measures, two-way ANOVA with Bonferroni post-hoc analyses were used. Behavioral and chemical panel analyses also used two-way ANOVA. For genechip analyses, the filtered genes (7922 probe sets) were tested statistically by two-way ANOVA (main effects of age and drug, as well as interaction) using the False Discovery Rate (FDR [55]) to gauge multiple testing error (see Results) and post hoc Fisher's Protected Least Significant Difference (PLSD) was used for all-pairwise comparisons among genes with significant main effects/interactions. For all statistical analyses, significance was considered present of p values were less or equal to 0.05.

## Results

### Chemical panel

**Figure 1:**
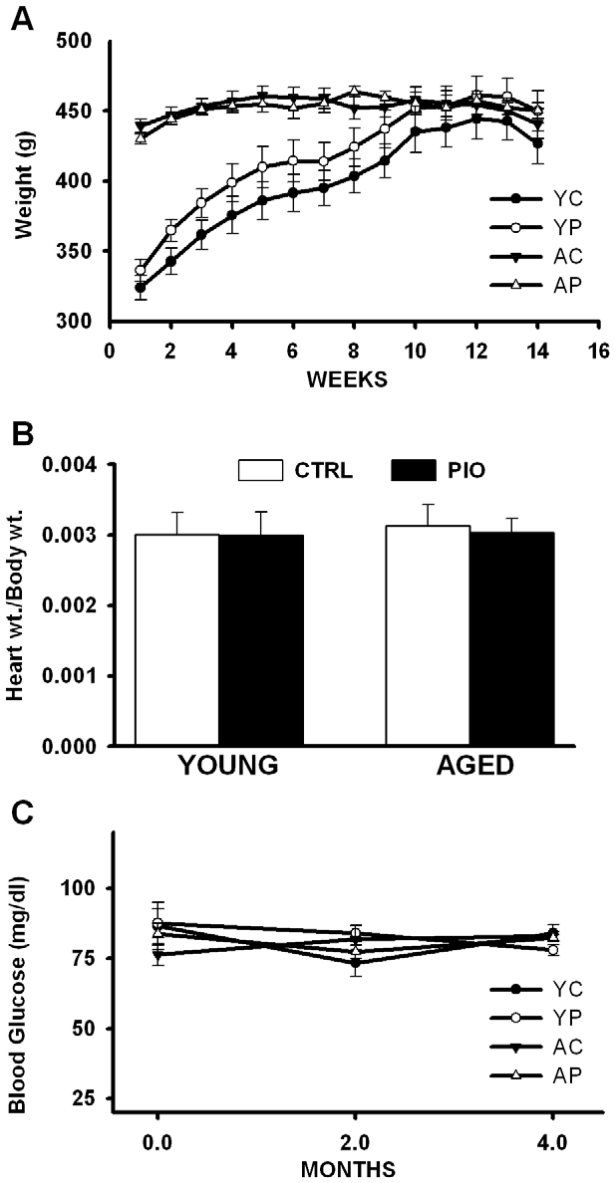
Growth curves, heart weights and blood glucose levels. (**A**) Body weights measured across age during the 14-week long study (YC = young control, YP = young PIO, AC = aged control and AP = aged PIO; 7–12 animals per group). (**B**) Heart weight normalized to body weight in 6–8 animals per group fed either control diet (CTRL) and PIO-enriched diet (PIO). (**C**) Blood glucose levels measured over the course of the study in 3–11 animals per group. Data represent mean ± SEM.

Analysis of blood serum obtained at time of hippocampal dissection for each animal, revealed a significant effect of PIO on lipids, including decreased total cholesterol (F(_1,25)_ = 4.46, p<0.05) and triglycerides (F_(1,25)_ = 13.9, p<0.001). Insulin levels also were significantly reduced by PIO in both age groups (F_(1,25)_ = 16.7, p<0.0005), consistent with similar human studies reported in the literature [56], [57], [58], [59], [60]. A significant age-dependent increase in HDL was seen (F_(1,25)_ = 5.26, p<0.05) but was not sensitive to PIO. *In vivo* glucose measures (from tail pricks) did not change with age or treatment during the course of the study (Fig. 1C), and analysis of sera obtained at time of dissection showed no glucose level change (see Table 1). Levels of alanine aminotransferase, a marker of liver health, were not affected by age or treatment. Interestingly, the triglyceride (TG) to HDL ratio (TG/HDL) was reduced to the same degree by PIO (∼50%) in young and aged animals (YC = 4.1; YP = 2.3; AC = 2.2; AP = 1.2), suggesting that our use of two PIO diets formulated at different drug concentration for the younger and older animals to compensate for different weights, had similar impact on peripheral lipids. Further, because this ratio is consider a surrogate marker for insulin resistance in humans, it seems PIO levels here where within a therapeutically-relevant range, reducing an indirect, yet classic clinical marker of insulin resistance. Therefore, in the F344 rat and at the dose tested, PIO provided significant reductions in lipid profiles and insulin levels, in a manner similar to that seen in clinical studies [56], [58], [60].

**Table 1:** Blood chemistry panel.

		YOUNG	AGED
**CHOL (mg/dl)**	**CTRL**	119.4±19.7	124.4±10.1
	**PIO**	85.4±8.0 *	108.1±5.9 *
**TG (mg/dl)**	**CTRL**	291.2±45.5	184.2±44.1†
	**PIO**	141.1±21.4 **	88.4±8.7 **,†
**HDL (mg/dl)**	**CTRL**	70.3±7.8	83.2±5.8†
	**PIO**	62.5±5.6	76.7±3.6†
**INSULIN (ng/ml)**	**CTRL**	7.2±1.8	6.1±1.6
	**PIO**	4.4±1.5 #	4.2±1.3 #
**GLUCOSE (mg/dl)**	**CTRL**	112.1±4.9	102.4±6.6
	**PIO**	109.5±8.2	101.4±8.1
**ALT (IU/L)**	**CTRL**	48.9±4.9	51.0±2.7
	**PIO**	37.5±3.2	47.7±4.6

Blood serum markers in control (CTRL) and PIO-treated (PIO) young and aged animals. Abbreviations: Cholesterol: CHOL; Triglycerides: TG; Alanine aminotransferase: ALT; High Density Lipoprotein: HDL.

*, **, and ^#^ indicate significant PIO effects (two-way ANOVA, p<0.05, p<0.001, and p<0.0005, respectively).

† indicates significant aging effect (two-way ANOVA, p<0.05). Data represent mean ± SEM in 7–8 animals per group.

### Organ and animal health

Animal weights were not different by age or treatment group by the end of the study, indicating younger animals on either the control or the PIO diet gained comparable weight (Fig. 1A). Because of prior reports that TZDs might be associated with adverse cardiovascular outcome [61], we measured heart weights in all animals. Normalized heart to body weight ratios showed no difference across groups (Fig. 1 B). Upon examination, no gross adiposity or cirrhosis was noted, and no internal organs showed distinguishable signs of pathology in the PIO group. Overall, it seems PIO was well-tolerated, with no observed anomaly detected in the animals' coat, eyes or skin, as well as internal organs, body, or heart weights.

### Dosage

PIO doses calculated from individual body weights and averaged food consumption (taken three times a week across the duration of the study) were 2.6±0.12 mg/Kg/day for the young PIO group and 1.9±0.07 mg/Kg/day for the aged PIO group. This difference was significant (Ttest, p<0.05).

### Behavioral characterization

**Figure 2:**
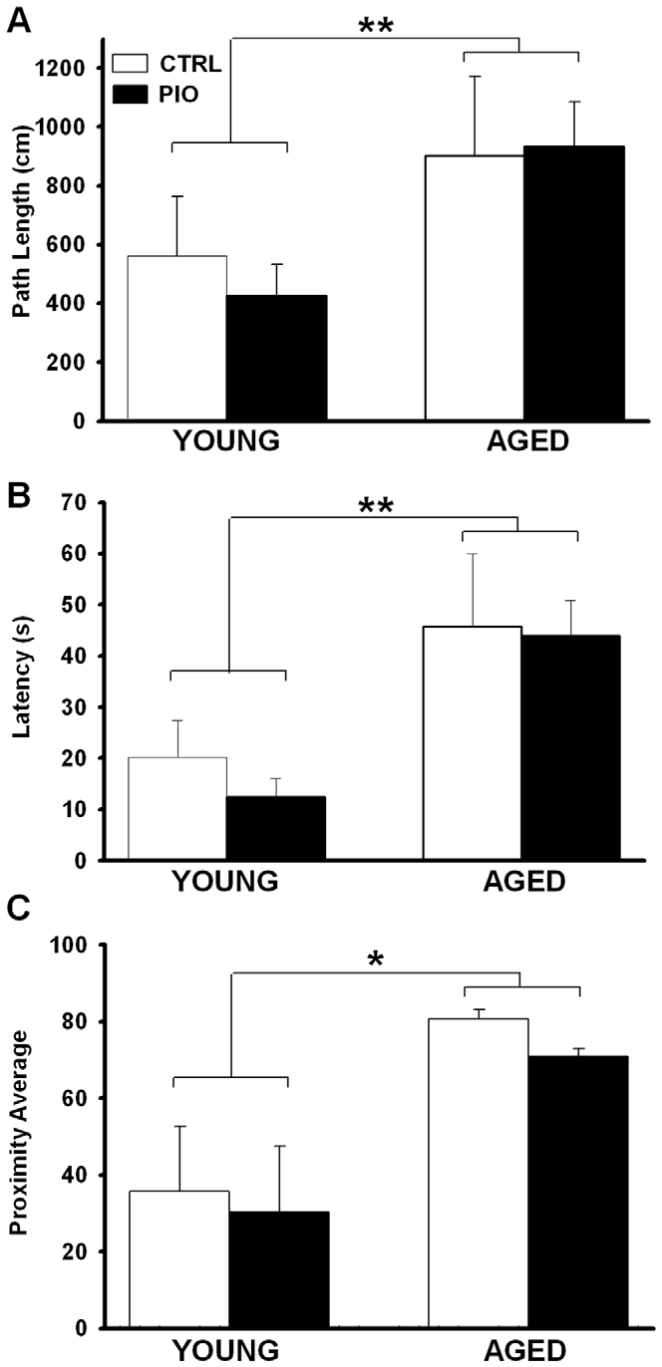
Spatial memory recall. (**A**) Path length to platform recorded across age and treatment groups (CTRL and PIO) show a significant increase in aged compared to young animals. (**B**) Latency to platform also was significant for age effect. In (**C**) Proximity averages representing a scalar index of recall shows a significant age effect. All measures represent animal performance on the probe day taken 24 h after the last training session. No PIO effects were noted. ** Indicates significant aging effect (two-way ANOVA, p<0.01), * indicates significant aging effect (two-way ANOVA, p<0.05). Data represent mean ± SEM in 4–8 animals per group.

Two-way ANOVA on conventional outcome measures associated with the Morris water maze (MWM) including path length and latency to platform, were tested for significance across the 4 days of training. No age or treatment differences were noted during this learning phase apart from a significant decrease in swim speed with age (F_(1,21)_ = 36.2; p<0.0001). Following the last day of training, a probe test (platform removed) assessed 24 h retention of platform location. Aged animals showed significantly longer path length to platform (F_(1,21)_ = 5.06, p<0.05) and latency to platform (F_(1,21)_ = 13.9, p<0.005), likely explained by a decrease in swim speed (F_(1,21)_ = 5.73, p<0.05). A proximal analysis which is not dependent on animals' speed or their original distance to the platform at the beginning of each trial [62] also showed that aged animals were swimming, on average, at a cumulative distance farther from the target than younger animals (F_(1,21)_ = 6.8, p<0.05). A proximity average scalar derived from the cumulative distance data divided by the latency to platform (Fig. 2C) also revealed significant age-dependent impairment on memory recall (F_(1,21)_ = 6.7, p<0.05). Thus, irrespective of the analysis used, no main effect of treatment was found, suggesting that at the doses tested, PIO could not reverse the age-dependent decrease in memory recall 24 h after the last training day.

### LTP

**Figure 3:**
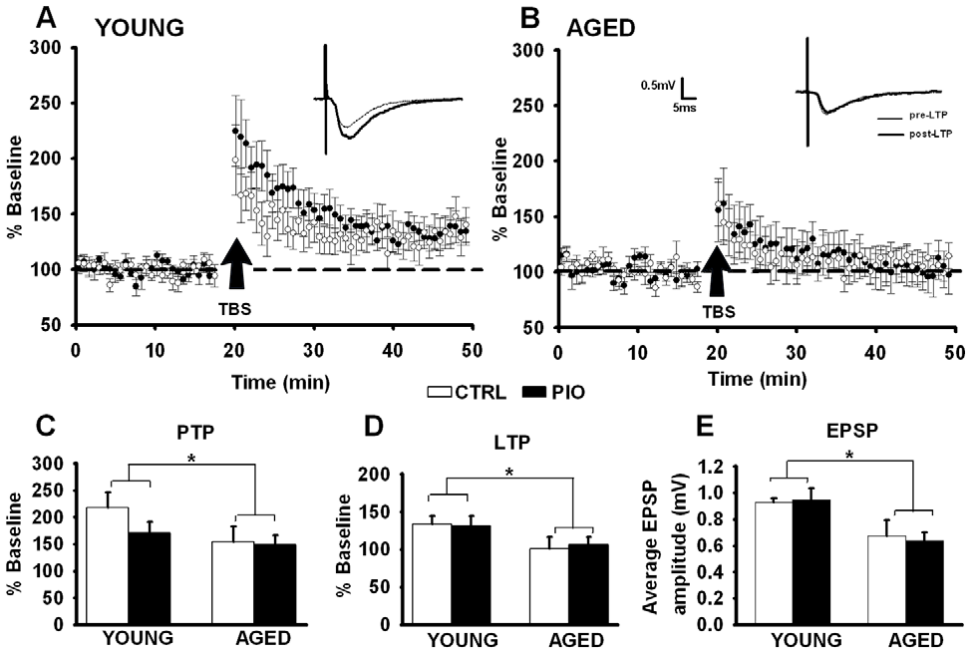
Theta-burst induced synaptic potentiation. (**A**) and (**B**) Normalized EPSP slopes measured across both age and treatment groups during baseline and following theta burst stimulation (TBS). Representative averaged EPSP traces for each age and treatment group are shown in insets. Post-tetanic potentiation (PTP) of the EPSPS was measured immediately following LTP induction (**C**). LTP maintenance was measure 25–30 min latter in CTRL and PIO-treated animals (**D**). (**E**) EPSP amplitudes taken before TBS reveal a significant effect of aging. No PIO effects were noted, * indicates significant aging effect (two-way ANOVA, p<0.05). Data represent mean ± SEM in 6–9 hippocampal slices from 3–5 animals per group.

As described previously [13], [41], [42], [43], [44], [45], [46], an age-dependent deficit in long-term potentiation (LTP) maintenance was seen 25–30 min following LTP induction (F_(1,26)_ = 6.82, p<0.02). Animals treated with PIO did not show signs of improvement on measures of LTP induction or maintenance (F_(1,26)_ = 0.21, p = 0.67). Similar results were seen on measures of post-tetanic potentiation (PTP) taken immediately following LTP induction, showing a significant main effect of age (F_(1,26)_ = 5.51, p<0.03), but no main effect of treatment (F_(1,26)_ = 0.80, p = 0.38). Figure 3 shows group means of normalized EPSP slopes across both age and treatment during 20 min baseline and for 30 minutes following LTP induction. Absolute measures of EPSP amplitudes prior to LTP induction are shown in Figure 3E and reveal a significant age-dependent decrease (F_(1,26)_ = 10.97, p<0.003) but no treatment effect (F_(1,26)_ = 0.01, p = 0.92). This result is consistent with prior evidence that at the CA1 synapse of aged animals, a decrease in the field EPSP is seen, likely mediated by a decrease in the number of functional synaptic contacts [12].

### AHP

**Figure 4:**
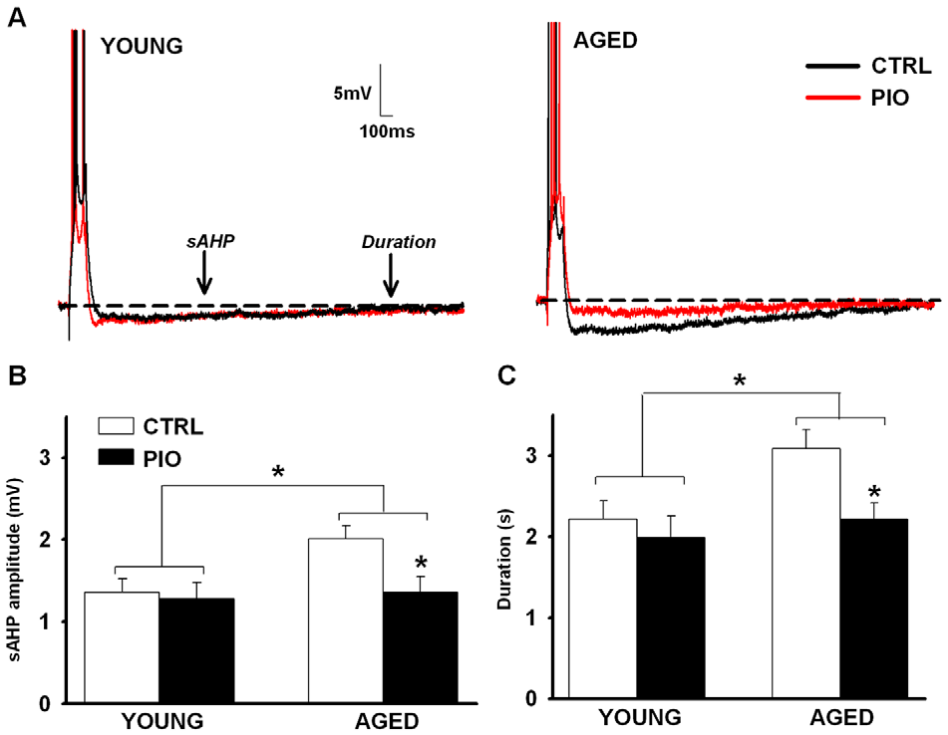
Measures of the Ca^2+^-dependent slow afterhyperpolarization (sAHP). (**A**) Representative examples of AHPs recorded in CA1 pyramidal cells from young (left) and aged (right) animals fed either control (CTRL) or PIO-enriched (PIO) diet. Both amplitude (**B**) and duration (**C**) of the sAHP were significantly enhanced in the aged group. In aged animals, however, long-term PIO treatment significantly reduced the sAHP (red traces in A). * indicates significant aging and PIO effects (two-way ANOVA, p<0.05). Data represent mean ± SEM in 12–21 recorded neurons from 6–7 animals per group.

Decreased excitability mediated by an increase in the sAHP is a reliable Ca^2+^-related biomarker of aging in hippocampal CA1 pyramidal neurons [63], [64], [65], [66], [67]. Two-way ANOVA on the AHP (Fig. 4) revealed A significant main effect of aging on measures of sAHP (Fig. 4) amplitude (32.5% increase over young, F_(1,56)_ = 3.96, p≤0.05; two-way ANOVA), and duration (26% increase over young, F_(1,56)_ = 5.05, p≤0.05; two-way ANOVA). Importantly, a main effect of PIO on these AHP measures also was noted with PIO causing a significant reduction in the AHP amplitude (F_(1,56)_ = 4.07, p≤0.05) and duration (F_(1,56)_ = 5.12, p≤0.05). PIO had a greater effect on the AHP recorded from the older animals, essentially reducing the AHP to levels seen in young animals (Bonferroni p≤0.05). No significant difference was found in the mAHP across the different age and treatment groups. No effects of PIO or aging on measures of neuronal health were noted (input resistances in MΩ, were 57.5±0.9 for young controls, 58.3±0.5 for aged controls, 52.1±0.4 for young PIO, and 57.3±0.9 for aged PIO), and all cells displayed overshooting action potentials.

### IR Signaling

**Figure 5:**
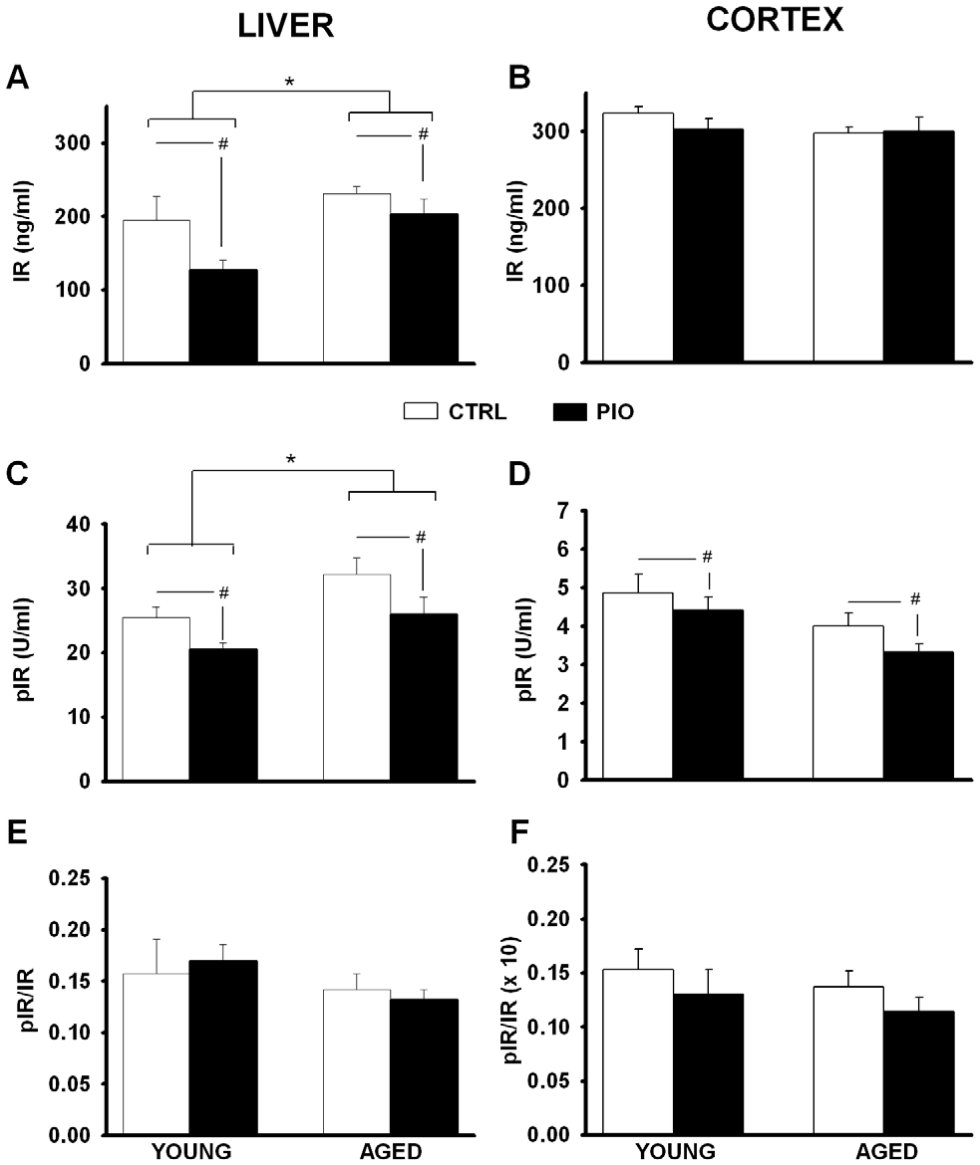
Insulin Receptor signaling. (**A**) and (**B**) Total insulin receptor levels (IR) were measured in liver (left) and cortex (right) as a function of age and diet (CTRL *vs.* PIO). In liver, the significant age-dependent increase in IR levels was significantly reduced by PIO treatment. (**C**) and (**D**) Phosphorylated IR levels (pIR) were also measured in liver (left) and cortex (right). While PIO treatment significant reduced pIR levels in both tissues and both age groups, only liver levels appeared to be sensitive to age. (**E**) and (**F**) Measures of signaling through IR, based on the ratio of pIR (C and D) to total IR (A and B) revealed no effects of aging or PIO treatment. * indicates significant aging effects (two-way ANOVA, p<0.05). ^#^ indicates significant PIO effects (two-way ANOVA, p<0.05). Data represent mean ± SEM in 5–8 samples per group.

Because PIO reduced insulin levels in the periphery (Table 1), we tested whether we could detect changes in insulin receptor (IR) signaling in the liver and cerebral cortex of each animal. We estimated the degree of IR signaling by normalizing phosphorylated to total IR (ratio) using two separate IR ELISA kits [68]. PIO significantly reduced phosporylated IR levels in both age groups (p<0.01; Fig. 5C). Quantitatively similar decreases in total IR also were seen (p<0.05; Fig. 5A), suggesting no net effect of age or treatment on liver IR signaling (Fig. 5E). Data from animal cortices also showed significant PIO-mediated decrease in phosphorylated IR levels (p<0.05; Fig. 5D), with no change in total IR (Fig. 5B). As surrogate indication of activated insulin receptor signaling, this reduction in phosphorylated IR in the brain and the periphery likely reflects PIO-mediated reduction in insulin levels. It is not clear, however, that this reflects decreases in insulin receptor signaling as neither ratios of phosphorylated to total IR signals (Figs. 5E and F), nor glucose levels, were significantly altered by age or treatment. Interestingly, while total IR levels were comparable in the liver and brain cortical tissues (Figs. 5A and B), phosphorylated IR levels were approximately 5 fold lower in the brain (Figs. 5C and D), suggesting lesser IR signaling in this tissue. Given that the animals in this study were non-diabetic, were not challenged with high fat diets, and were not pathologically aged, these results indicate that reducing insulin levels in healthy animals seems to have had little impact on insulin signaling.

### Gene Microarrays

**Figure 6:**
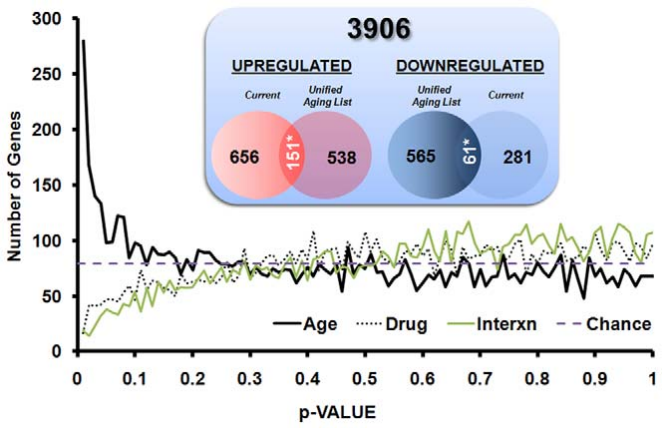
Hippocampal microarray analyses and comparisons to prior aging studies. P-value histogram derived from the results of the two-way ANOVA (age and drug) reveals robust aging, but not drug or interaction effects. Changes in hippocampal microarray gene signatures were separated according to main effects of age, drug, or interaction (age×drug). While more genes than expected by chance (dashed horizontal line) were shown to be age-sensitive (819, bold line), fewer than expected by chance were found to be PIO-sensitive (189, dotted line), or associated with the interaction (125, gray line) (median FDRs for age = 0.21; for drug = 2.3; for interaction = 3.8). Dataset was derived from hippocampal tissue isolated from 6–8 animals per group. **Inset:** Agreement of age-related hippocampal gene expression across this and prior studies. Genes found to be present and annotated across four studies (3906) were subjected to overlap analysis for common age-related transcriptional profiles. Unified aging list includes genes found to change three studies [47], [48], [50]. Here, the number of genes expected to overlap by chance (94 up, 36 down) was significantly greater (151 up and 61 down; p<0.01). * indicates p<1.7E-8 for up and p<2.7E-4 for downregulated genes.

Two-way ANOVA applied to the 7922 genes in the filtered list (see Methods) yielded three sets of p-values (main effects of aging and drug treatment, and interaction). Because each of these sets of p-values is vulnerable to the error of multiple testing, we plotted p-value frequency histograms (Fig. 6) for each set of p-values, and superimposed the number of findings expected by chance. Interestingly, both drug and interaction terms performed well below chance (at p≤0.05; FDR>2.3), while the main effect of age showed a strong, reliable signature (at p≤0.05; FDR = 0.21), similar to that seen in prior studies [48], [49], [51], [70], [71]. Thus, microarray-based transcriptional signatures of hippocampal tissue in this study reliably detected aging-related gene signatures comprised most notably of increased inflammatory pathways. Specifically, age-dependent increases in IL-18, IL-33, and IL-10 (receptor binding unit), and an age-dependent decrease in IL-16 were seen (for a complete list of genes significantly different with age, see Table S1). However, no significant ‘PIO’ or ‘age×PIO’ interaction effects were noted, possibly because the drug has no effect on hippocampal transcription, did not penetrate the CNS, or, alternatively, because the study was underpowered for the discovery of those changes.

To test whether the effects of PIO may have been limited to targeted pathways or processes that may have been lost in the overall non-significant PIO or age×PIO interaction effects reported in our gene microarray analysis (two-way ANOVA, Fig. 6), we interrogated the Gene Ontology (GO; www.geneontology.org) database of biological processes. Two highly relevant GO terms related to PIO's mechanism of action, namely, insulin receptor signaling pathway (GO:0008286) and inflammatory response (GO:0006954) were isolated. Within these pathways, gene symbols associated with GO, annotated to rat, and present on our microarray platform were identified (total genes: insulin receptor signaling = 37; inflammatory signaling = 141). We next determined if any of the genes in these pathways were significantly changed, based on either the main effect of drug, or on the interaction term (p≤0.05) from the two-way ANOVA. Insulin signaling genes were significant by neither main effect of drug nor by interaction. Among inflammatory response genes, 3 were significant by the main effect of drug (Ednra- decreased; Serpina3n- decreased; Zfp36- upregulated), and 3 more were significant by interaction (F3 and Mug1- PIO suppresses an age-related increase in expression, Tlr4- PIO suppresses expression in young subjects only). We then evaluated the likelihood that, by chance, 3 genes would appear significant out of 141 by either interaction or main effect of drug ANOVA p-values. To test this, we conducted a resampling analysis (1000 iterations of 141 randomly sampled genes) and fit normal distributions to the resulting frequency histograms (data not shown). Based on this approach, neither 3 genes in the interaction term (p = 0.27), nor 3 genes in the main effect of drug (p = 0.54) were found to be significant, inspiring little confidence in PIO's ability to manipulate these targeted pathways. In contrast, using this approach, aging effects on inflammatory genes were highly significant (p = 0.01). Thus, although it is possible that some genes within these pathways are changed due to the effect of PIO, the effect does not appear either statistically reliable or pathway-selective.

The overall main effect of age on microarray signatures, however, was clearly detectable and was subjected to further analysis. Because the tissue collection procedure here (ventral and dorsal hippocampal quarters from both hemispheres are pooled for each animal- see Methods) has not been used in other studies, we tested to see whether this strong, aging-related transcriptional profile agreed with prior aging-related transcriptional profiles in F344 rat hippocampus (Fig. 6 inset). Using three prior hippocampal aging studies [47], [48], [50], we created a consolidated list of 3906 total genes that were present in all three prior studies as well as in the present study. 656 total genes were significantly upregulated and 565 downregulated in at least one of the prior three studies. In the current data set, 538/3906 were upregulated and 281/3906 were downregulated. Thus, of the 656 upregulated genes identified in prior studies, 538/3906 (or 13.8%; 74 genes) should agree by chance alone. In fact, 151 agree and this is highly significant (p = 1.04^−10^, binomial test). Similarly, for downregulated genes, 281/3923 significant in the present study suggests that 7.2% (or 40 genes) should agree by chance. Again, 61 genes were found in the overlap and this was significant (p = 6.3^−4^, binomial test). Although downregulated genes were significantly overrepresented, the magnitude of the effect was relatively small (61 *vs.* 40 expected by chance). DAVID analysis of aging changes (Table 2) showed downregulated pathways that are often associated with reduced neuronal activity (e.g., Ca^2+^ binding, receptor signaling, vesicle, and secretion), and reduced glucose-driven energy production (e.g., phosphorylation, ATP synthesis). Upregulated pathways were strongly associated with increased inflammatory signaling (e.g., antigen processing, response to wounding, adaptive immune response, and leukocyte cytotoxicity) and lipid/membrane processes possibly reflecting a metabolic shift towards fatty acid β oxidation and/or compensatory re-myelination (e.g., lysosome, myelination, membrane lipid catabolism, and lipid synthesis). As with the genes themselves, these age-related up- and down-regulated processes, particularly increased inflammatory signaling and decreased neuronal activity, are highly similar to findings from previous microarray studies of rodent hippocampal aging [47], [48], [50], [69], [70].

**Table 2:** Pathway analysis for aging-related genes.

***Downregulated***
**Ca^2+^ ion binding** (# 17, p = 0.0035)- Actn1, Ap1gbp1, Calb1, Camk4, Clstn1, Dag1, Dmp1, Gpd2, LOC684520, Ncald, Nell2, Pcdha1, Rnf111, Scg2, Slc24a3, Syp, Tesc
**Receptor activity** (# 27, p = 0.0008)- Acvr2a, Atrnl1, Chn1, Derl1, Epha7, Gabra5, Gabrb3, Gpr176, Grm7, Htr1b, Il22ra2, Insr, LOC683548, Mmd, Nr3c2, Oprm1, Pcdha1, Pcsk5, Ptpro, Ptpru, Ring1, Slc7a1, Sra1, Sstr2, Strap, Tfrc, Trpc5
**ATP synthesis coupled H^+^ transport** (# 06, p = 0.0056)- Atp5a1, Atp5h, Atp6ap1, Atp6v0e2, Atp6v1b2, Atp6v1c1
**Tyrosine phosphorylation of Stat3** (# 04, p = 3.7^−06^)- Clcf1, Il22ra2, Ppp2ca, Ppp2cb
**Vesicle** (# 17, p = 0.0145)- Abca3, Agtr1a, Agtrl1, Ap1gbp1, Capza2, Chgb, Copb1, Kif3a, Rab12, Rab3d, Scamp1, Scg2, Syn1, Syp, Syt17, Tfrc, Trim9
**Phosphorylation** (# 27, p = 0.0039)- Acvr2a, Atp5a1, Atp5h, Atp6ap1, Atp6v0e2, Atp6v1b2, Atp6v1c1, Camk4, Cask, Clcf1, Dclk1, Epha7, Ikbkap, Il22ra2, Insr, LOC687516, Map2k1, Map2k5, Map3k12, Mark3, Nme1, Pcsk5, Plk2, Ppp2ca, Ppp2cb, Prpf4b, Uhmk1
**Secretion** (# 18, p = 0.0272)- Agtr1a, Arf5, Capza2, Copa, Copb1, LOC498353, Nr3c2, Osbpl5, Rab3d, Sar1a, Scamp1, Scg2, Scrn1, Sec22a, Snca, Syn1, Trim9, Yipf5
***Upregulated***
**Lysosome** (# 24, p = 4.8^−08^)- Abca2, Aga, Cd74, Ctsd, Ctss, Dnase2a, Fnbp1, Fuca1, Gm2a, Gusb, Hexa, Hexb, Ifi30, Lamp1, Lamp2, Laptm5, Lgmn, Neu1, Nppa, Ppt1, Psen1, Slc15a3, Tpp1, Trip10
**Antigen processing and presentation** (# 11, p = 6.7^−09^)- B2m, Btnl3, Cd74, Ctse, Fcgr2b, Ifi30, Psmb8, Psme2, RT1-Aw2, RT1-Ba, RT1-M3
**Response to wounding** (# 39, p = 6.5^−06^)- Aif1, Alox5ap, Apod, Ass1, C1qa, C1qb, C1qc, C3, Ccl5, Cd9, Cfh, Ctgf, Cxcl14, Dsp, Ednrb, Entpd2, Fcgr2b, Gatm, Gfap, Gsn, Il10rb, Itgb2, Klk6, Lta4h, Neu1, P2ry12, Pllp, Ptafr, Ptpn6, Pycard, Rab27a, RT1-Aw2, S100a9, Serpina1, Srprb, Stat3, Tbxas1, Tgfa, Tm4sf4
**Adaptive immune response** (# 12, p = 4.2^−07^)- C1qa, C1qb, C1qc, C3, Cd74, Fcgr2b, Il18, Inpp5d, Irf7, Pirb, Ptprc, Rab27a
**Actin filament** (# 06, p = 0.0014)- Actc1, Aif1, Cnn3, Lcp1, Myo9b, Wipf1
**Leukocyte mediated cytotoxicity** (# 03, p = 0.0116)- Fcgr2b, Ptprc, Rab27a
**Eicosanoid metabolic process** (# 07, p = 0.0011)- Alox5ap, Cd74, Lta4h, Pla2g4a, Ptgds2, Ptgs1, Tbxas1
**Glucosamine metabolic process** (# 04, p = 0.0337)- Chi3l1, Hexb, Nagk, Renbp
**Myelination** (# 07, p = 0.0334)- Aspa, Cd9, Hexa, Hexb, Klk6, Pllp, Srprb
**Membrane lipid catabolic process** (# 04, p = 0.0067)- Gm2a, Hexa, Hexb, Pla2g4a
**Lipid biosynthetic process** (# 18, p = 0.0498)- Acsl3, Agt, Alox5ap, Cd74, Cyb5r2, Fdft1, Ggps1, Hexb, Hspc105, Lta4h, Nr0b1, Pla2g4a, Ptgds2, Ptgs1, Srd5a1, Srebf1, Stard3, Tbxas1
**Regulation of neuron apoptosis** (# 08, p = 0.0084)- Agt, Aif1, Dlx1, Nqo1, Ppt1, Psen1, RT1-Aw2, Tgfa

Lists of significantly (p≤0.05, two-way ANOVA main effect of age) down- and up-regulated genes were subjected to overrepresentation analysis using DAVID's clustering function (Methods). Representative pathways from each cluster found to have significantly more genes than expected by chance (p≤0.05, modified Fisher's exact test) are shown. The pathway (bold) is followed in parentheses by the number of genes found to be significant in that pathway (#) and the likelihood that such a number could be found by chance (p = ). This is followed by a list of the gene symbols within that pathway.

### Pro-inflammatory Markers

Peripheral inflammation was monitored using a serum-based multiplex bead assay targeted for key cytokines, namely Interleukin (IL)-1β, IL-6 and tumor necrosis alpha (TNF-α). Of the twenty-nine serum samples analyzed, two outliers (one YC and one AP) were removed from the analysis for having signals twice the standard deviation associated with the mean of that group. ANOVA analysis of the peripheral inflammatory markers on the remaining 27 animals (n = 6 young control-YC; n = 8 young PIO-YP; n = 7 aged control-AC; n = 6 aged PIO-AP) showed an age-dependent trend toward larger IL-6 signals, with a near doubling in signal intensity (F_(1,24)_ = 2.7, p = 0.1), however, no PIO effects were detected (p = 0.70). IL-6 signals for each group were (in ρg/mL): 122.8±16.7 for the YC, 142.8±26.9 for the YP, 204.9±36.9 for the AC, and 165.3±40.7 for the AP. Serum signals from TNF-α and IL-1β were below detectable levels (<24.4 ρg/mL). Compared to the microarray signatures, the results in the periphery showed only modest increases in inflammatory cytokines with age but also reveal that at the concentration tested, PIO did not significantly reduce inflammation in either tissue.

## Discussion

We investigated pioglitazone's potential role in reducing peripheral and central markers in a well-characterized animal model of cognitive decline with aging. We chose the antidiabetic drug because of its favorable permeability into the CNS (∼10%, [71]), positive effects in the treatment of type 2 diabetes mellitus (T2DM), and its reported success in the treatment of neurodegenerative disease in several animal models. Choice of animal age, dose, and delivery method were selected to mimic clinical conditions associated with the treatment of late-onset diabetes. In prior studies on the impact of the TZD in the brain, higher doses have been used, and often in more pathological models, including Alzheimer's, Parkinson's disease, and spinal cord or brain injury. These models are characterized by pathological processes that are likely present in normal aging, but to a lesser extent. As such, and because of the reported beneficial use of TZDs in alleviating cognitive decline in elderly patients with or without diabetes, we felt it important to test PIO's role in an animal model of normal aging. We focused on several well-established brain biomarkers of aging including learning and memory and inflammatory processes.

Several lines of evidence show that specific TZDs can have direct effects on Ca^2+^ homeostasis, particularly L-type voltage-gated Ca^2+^ channels in cardiovascular tissues [72], [73], [74], [75], [76], [77], as well as in neurons [31], [32]. Given that Ca^2+^ dysregulation is considered a hallmark of brain aging [65], [78], [79], [80], is present in animal models of diabetes [81], [82], [83], [84], and appears to contribute to insulin resistance in the periphery [85], we tested the hypothesis that PIO might reduce Ca^2+^-dependent brain biomarkers of aging *in vivo* and thereby provide some relief from the cognitive decline seen with aging. At the dose and duration tested, PIO did not alleviate age-dependent memory decline (Fig. 2), nor did it improve LTP maintenance (Fig. 3). This was surprising given PIO's effect on the Ca^2+^-dependent slow after-hyperpolarizing potential (Fig. 4). Indeed, it is well-recognized that reducing the AHP increases cellular excitability and can improve cognitive performance (reviewed in [12], [67], [78]). In prior *in vitro* studies, we have shown that PIO can reduce NMDA-mediated currents and associated intracellular Ca^2+^ levels [31], and it is possible that the beneficial effects of reducing the AHP with age in the current study were masked by concomitant decreases in NMDA-mediated signaling. However, because the effects of PIO on NMDA currents were determined in neurons *in vitro*, future studies focusing on whether long-term *in vivo* PIO can have inhibitory effects on NMDA currents in hippocampal slices will help to address this issue. With respect to the effect of PIO on the AHP, one possibility is that it is mediated by an indirect action on glucocorticoid regulation. In fact, in AD models, TZDs have been shown to improve cognition via alterations in glucocorticoid signaling [26], [86], and there is good evidence that the AHP is sensitive to glucocorticoids [87].

We measured insulin levels as well as insulin signaling in the brain and the periphery of young and aged animals. Our results show that insulin and glucose levels were not elevated with age, but insulin levels were significantly reduced by PIO in both age groups (Table 1). Given that our animals were not diabetic, we predicted that a decrease in insulin levels would result in a concomitant reduction in insulin receptor signaling, at least in the periphery and perhaps also in the brain. ELISA assays on liver (peripheral) and cortical (central) tissues showed that while the ratio of phosphorylated to total insulin receptor did not change, PIO did cause a significant reduction in phosphorylated insulin receptor levels (Fig. 5). These results suggest that insulin receptor levels exist in a dynamic insulin-sensing equilibrium both in the brain and the periphery. It is not clear, however, whether the fewer total receptors remaining might be more efficient at translating insulin's action through differential regulation of downstream targets of the insulin receptor, including IRS-1, Pi3K, Akt, and SH2 [88]. Further, it is also likely that other insulin sensitive tissues (e.g., muscle or fat) could show enhanced sensitivity to PIO's effects on insulin, resulting in more robust changes in insulin receptor signaling. Because insulin levels were decreased in both young and aged animals treated with PIO, and AHP reductions were only seen in the aged group, we do not believe insulin levels directly influenced the AHP under the condition tested. There is, however, evidence supporting a decrease in insulin sensitivity in the brain of AD patients and in AD models [89], [90], [91], and we are currently testing this hypothesis in our aging model. However, it is unclear whether the aging F344 rat is a good model of T2DM, and responds to high fat diets with signs of insulin resistance in both the periphery and the brain [92], [93]. Here, therefore, we believe that PIO's effects on insulin signaling were somewhat blunted because animals were healthy and non-diabetic. On the other hand, one would predict that under conditions more representative of human aging, where accumulated exposure to high fat diets and a sedentary life style contribute to T2DM, PIO might significantly reduce insulin levels [56], [57], [58], [59], increase insulin sensitivity, and likely increase insulin signaling.

Prior studies in CNS and peripheral cell types modeling trauma and insult (e.g., LPS, PMA) demonstrate that PPAR-γ activators play a critical role in reducing inflammatory cytokines (interleukin-6, interleukin 1-β and TNF-α), including activation of inducible nitric oxide synthase (iNOS) [94], [95], [96], [97], [98], [99]. Similarly, in animal models of ischemia, stroke, hypertension, stress, and Parkinson's disease, which are also characterized as pro-inflammatory conditions, PPAR-γ agonists provide significant neuroprotection [100], [101], [102], [103], [104], [105], [106], [107], [108], [109], [110], [111], [112]. In AD animal models also, PPAR-γ agonists appear to reduce baseline inflammatory levels [24], [26], [113], [114]. Only one prior animal study examined the effects of TZDs under non-pathological aging conditions. The authors reported that the increase in proinflammatory cytokine levels (IL-1β) in the hippocampus of aged F344 rats was not sensitive to the actions of the TZD rosiglitazone (10 mg/Kg/day) yet the drug caused significant improvement in contextual fear conditioning [115]. Similarly, a prior publication using 20 mg/Kg/day PIO for four months in transgenic mouse models of AD (Tg2576) revealed very little anti-inflammatory effects of PIO (based on microglial activation, soluble Aβ levels, and plaque burden) when compared to ibuprofen treatment [114]. The same group, however, convincingly showed that a 40 mg/Kg/day PIO dose could significantly reduced brain inflammation in the APPV717I transgenic mouse [24]. Here, therefore, we examined inflammatory cytokines in the serum, and inflammatory signaling in the brain using hippocampal microarray analyses in the context of normal aging, and at low doses of PIO. While a clear inflammatory signature was present in the brain as previously reported in microarray studies of aging [11], [47], [48], [50], [116], [117], [118], [119], [120], PIO did not significantly reduce inflammatory markers in the hippocampus. In the periphery, no robust age-dependent change in inflammatory cytokine levels was seen (although a trend in increased IL-6 levels was noted in aged animals), precluding an effect of PIO. Importantly, reductions in peripheral insulin and lipids indicate the target therapeutic window for PIO was reached. Under these conditions, thus, it is unclear that PIO was able to reduce central and peripheral inflammatory markers in an animal model of aging, and together, these results suggest that higher PIO doses might be necessary to reduce inflammatory pathways and exert beneficial cognitive effects.

Using an unbiased microarray analysis approach on hippocampal tissue, our study compared the effects of a brain permeant TZD treatment in younger and older animals, and showed that while age-dependent gene signatures were clearly present at both the gene (Fig. 6) and pathway (Table 2) levels, PIO effects on gene expression were virtually absent. Possible reasons for this include: low statistical detection power, insufficient drug exposure, or lack of influence of this treatment regimen on hippocampal transcription. Low statistical power is a possibility; however, the treatment main-effect histogram dips well below chance at small p values (Fig. 6), and it seems unlikely that increasing the number of subjects would allow us to detect significant transcriptional effects of PIO. Regarding drug exposure, PIO reduced peripheral blood chemistry measures significantly and in the direction predicted by prior work (see discussion above and Table 1), suggesting that treatment levels were appropriate. Thus, it seems reasonable to conclude that PIO did not exert a detectable transcriptional effect on hippocampal gene transcription, and that this lack of central influence may be due to either reduced blood-brain barrier penetration or a frank lack of response from hippocampal tissue. Nevertheless, because PIO has established effects involving insulin and inflammatory processes, we also directly investigated genes associated with these processes and tested their sensitivity to PIO in the brain. Although it is not possible to evaluate the biological importance of the 6 genes that were identified without functional genetic manipulation (e.g., knock-in), our resampling analysis revealed that these targeted pathways were not statistically significant. It is interesting to note, however, that of the six genes identified, one of them (Tlr4) recently was found to be sensitive to PIO in monocytes and macrophages [121], reinforcing the role of PIO as an anti-inflammatory agent, and suggesting Tlr4 might be a common target of PIO in peripheral and central tissues.

Compared to prior aging studies, our overlap analysis (Fig. 6 inset) suggests that, irrespective of where along the dorsal-ventral axis it is sampled, the hippocampus shows increased inflammatory markers with age, and validates the use of hippocampal extremities in future microarray studies. Interestingly, upregulated inflammatory categories, historically the most consistent and largest magnitude of the aging brain transcriptional signatures, remain largely unperturbed by PIO administration.

To our knowledge, this is the first study testing long-term PIO treatment in the F344 rat with age, specifically investigating whether a commonly prescribed drug may have off-target cognition enhancing effects in a rat model of aging. The study was designed using a clinically-relevant dose and delivery method. As expected, several signatures of aging were present in older animals, characterized by weak peripheral and robust central inflammatory increases, reduced spatial memory and LTP maintenance, and increased Ca^2+^-dependent AHPs. Peripheral insulin levels, phosphorylated insulin receptors in the CNS and the periphery, and the AHP were significantly reduced by PIO. While the mechanism through which PIO may mediate its central effects (AHP reduction, reduced phosphorylated insulin receptor) is not clear, it does not appear to occur via a transcriptional process. Given the increased incidence in metabolic syndrome and T2DM seen in the aging population, together with the high numbers of prescriptions written for TZDs, our study has direct therapeutic relevance and suggests future experiments testing the use of these agents at clinically-relevant doses for the treatment of neurological or cognitive conditions are needed. Nevertheless, the results of our study do not preclude the beneficial effects of TZDs in the elderly where metabolic dysregulation and diabetes are often reported.

## Supporting Information

Table S1 (5.20 MB XLS)
